# Comparison of the Abilities of SD-OCT and SS-OCT in Evaluating the Thickness of the Macular Inner Retinal Layer for Glaucoma Diagnosis

**DOI:** 10.1371/journal.pone.0147964

**Published:** 2016-01-26

**Authors:** Kyoung Min Lee, Eun Ji Lee, Tae-Woo Kim, Hyunjoong Kim

**Affiliations:** 1 Department of Ophthalmology, Seoul National University College of Medicine, Seoul National University Bundang Hospital, Seongnam, Korea; 2 Department of Applied Statistics, Yonsei University, Seoul, Korea; Duke University, UNITED STATES

## Abstract

**Purpose:**

To compare the abilities of spectral-domain optical coherence tomography (OCT) (SD-OCT; Spectralis, Heidelberg Engineering) and swept-source OCT (SS-OCT; DRI-OCT1 Atlantis system, Topcon) for analyzing the macular inner retinal layers in diagnosing glaucoma.

**Methods:**

The study included 60 patients with primary open-angle glaucoma (POAG) and 60 healthy control subjects. Macular cube area was scanned using SD-OCT and SS-OCT on the same day to assess the thicknesses of the macular retinal nerve fiber layer (mRNFL), ganglion cell layer plus inner plexiform layer (GCIPL), and total retinal layer in nine subfields defined by the Early Treatment Diabetic Retinopathy Study (ETDRS). The abilities of the parameters to discriminate between the POAG and control groups were assessed using areas under the receiver operating characteristic curves (AUCs).

**Results:**

Glaucoma-associated mRNFL and GCIPL thinning was more common in the outer zones than inner zones for both SD-OCT and SS-OCT. The mRNFL and GCIPL measurements showed distinct pattern differences between SD-OCT and SS-OCT in each ETDRS subfield. Although the glaucoma-diagnosis ability was comparable between SD-OCT and SS-OCT for most of the parameters, AUC was significantly larger for SD-OCT measurements of the GCIPL thickness in the outer temporal zones (*p* = 0.003) and of the mRNFL thickness in the outer nasal zones (*p* = 0.001), with the former having the largest AUC for discriminating POAG from healthy eyes (AUC = 0.894).

**Conclusion:**

Spectralis SD-OCT and DRI SS-OCT have similar glaucoma-diagnosis abilities based on macular inner layer thickness analysis. However, Spectralis SD-OCT was potentially superior to DRI SS-OCT in detecting GCIPL thinning in the outer temporal zone, where the glaucomatous damage predominantly occurs.

## Introduction

Glaucoma is characterized by the progressive degeneration of retinal ganglion cells (RGC) and the loss of their axons [1,2]. Evaluating the number of RGC axons lost using optical coherence tomography (OCT) has been essential for diagnosing glaucoma and monitoring its progression [3–5]. In recent decades the peripapillary retinal nerve fiber layer (RNFL) has been a major focus in OCT-based glaucoma evaluations [4–7]. However, recent advances in OCT technology have enabled more detailed segmentation of the macular inner retinal layers, and thus allowed the quantitative evaluation of macular RGC damage [8–10]. Studies have shown that the thickness of the macular inner retinal layer as measured using spectral-domain OCT (SD-OCT) is useful for the diagnosis of early glaucoma [11–14] and for evaluating glaucoma progression [15–17]. In addition, potential advantages of macular analysis have also been suggested in patients with high myopia [18,19], parafoveal scotoma [20,21], and advanced disease [16,22].

New software that allows the segmentation of individual layers of the retina has been recently designed for the Spectralis OCT system (Heidelberg Engineering, Heidelberg, Germany) [23]. This enables the independent measurement of each of the retinal layers of the macula, including the macular RNFL (mRNFL) and ganglion cell layer (GCL). The usefulness of this algorithm in discriminating between glaucoma suspects and healthy subjects has recently been evaluated [23]. However, the glaucoma-diagnosis ability of this new algorithm has yet to be reported.

On the other hand, a newer generation swept-source OCT (SS-OCT) system, called the DRI-OCT1 Atlantis system (Topcon, Tokyo, Japan), also has an automated algorithm for segmenting macular inner retinal layers, and provides the thicknesses of the mRNFL and the GCL plus inner plexiform layer (IPL) (GCIPL) in the macular area. Since SS-OCT penetrates deeper into the tissue and provides high-resolution images [24,25], the ability to measure the macular inner layer thicknesses using SS-OCT is also of interest in the diagnosis of glaucoma. It was found recently that the diagnostic ability was similar between measuring the RNFL thickness over the macular and peripapillary area using DRI SS-OCT and measuring the circumpapillary RNFL (cpRNFL) thicknesses using DRI SS-OCT or Spectralis SD-OCT [26]. More recently, the diagnostic abilities of DRI SS-OCT and Cirrus SD-OCT (Carl Zeiss Meditec, Dublin, CA, USA) in measuring the macular GCIPL thicknesses were also found to be similar [27]. However, the usefulness of DRI SS-OCT assessments of macular inner retinal layers in diagnosing glaucoma relative to using Spectralis SD-OCT has not been fully evaluated.

The macular segmentation software programs provided with the Spectralis OCT and DRI-OCT1 Atlantis systems both have a sectorial thickness evaluation tool using the circle defined by the Early Treatment Diabetic Retinopathy Study (ETDRS), which allows a 1:1 comparison of the thickness in each sector between the two OCT systems. The present study aimed to determine and compare the glaucoma-diagnosis abilities of macular inner retinal layer analysis performed using SD-OCT and SS-OCT with the Spectralis OCT and DRI-OCT1 Atlantis systems, respectively.

## Materials and Methods

### Participants

This prospective, cross-sectional study included patients with primary open-angle glaucoma (POAG) and age-matched healthy controls. Written informed consent to participate was obtained from all subjects, and the study protocol was approved by the Seoul National University Bundang Hospital Institutional Review Board and followed the tenets of the Declaration of Helsinki.

Subjects for the POAG and healthy control groups were enrolled from patients who visited Seoul National University Bundang Hospital Glaucoma Clinic between September 2014 and April 2015. All of the participants underwent comprehensive ophthalmic examinations that included assessment of visual acuity, Goldmann applanation tonometry, refraction tests, slit-lamp biomicroscopy, gonioscopy, and dilated stereoscopic examination of the optic disc. The following investigations were also performed: central corneal thickness measurement (Orbscan II, Bausch & Lomb Surgical, Rochester, NY, USA), axial length measurement (IOLMaster version 5, Carl Zeiss Meditec, Dublin, CA, USA), color fundus photography and red-free fundus photography (EOS D60 digital camera, Canon, Utsunomiyashi, Japan), SD-OCT (Spectralis OCT) scanning of the circumpapillary and the macular cube area, SS-OCT (DRI-OCT1 Atlantis system) scanning of the macular cube area, and standard automated perimetry (Humphrey Field Analyzer II 750, 24–2, Swedish interactive threshold algorithm, Carl Zeiss Meditec).

All participants had to meet the following inclusion criteria: a best-corrected visual acuity of greater than 20/40, a spherical equivalent ranging from –8.0 to +6.0 diopters, cylinder correction within –3.0 to +3.0 diopters, and the presence of an open angle as confirmed by gonioscopy. The exclusion criteria were retinal or neurologic diseases that could cause visual field defects, history of intraocular surgery other than uneventful cataract surgery, unreliable visual field tests (fixation loss rate >20%, or false-positive or false-negative error rates >25%), and poor-quality SD-OCT and SS-OCT images that did not allow the precise segmentation of each inner retinal layer. When both eyes were eligible, one eye was randomly chosen for data analysis.

POAG was defined as the presence of glaucomatous optic nerve damage (neuroretinal rim notching or thinning, or presence of an RNFL defect), an open angle and associated visual field defects without ocular disease or conditions that may elevate the intraocular pressure (IOP). A glaucomatous visual field defect was defined as (1) outside the normal limits on a glaucoma hemifield test; or (2) three contiguous nonedge points (allowing for the two nasal-step edge points), with a probability of <5% of being normal and one with a probability of <1% based on pattern deviation; or (3) a pattern standard deviation of <5%. Those visual field defects were confirmed on two consecutive reliable tests (fixation loss rate of ≤20%, and false-positive and false-negative error rates of ≤25%).

Healthy subjects were defined as those having an IOP of <21mmHg, a normal-appearing optic disc, and normal visual field results. The visual field was considered normal when there was no glaucomatous visual field defect or neurological defect.

### cpRNFL and macular inner retinal layer analysis using SD-OCT and SS-OCT

The SD-OCT and SS-OCT images of the macular area were obtained on the same day. The cpRNFL was scanned using the circular scan mode of the Spectralis OCT system, which consisted of 768 A-scans. The scan circle subtended 12°, and the diameter in millimeters depended on the axial lengths.

The volumetric scanning of the macular area was performed using both SD-OCT and SS-OCT for each subject. For SD-OCT, 61 B-scan sections parallel to the foveo-disc axis were obtained in the perifoveal area covering 8.9 mm × 7.4 mm. Each section had nine OCT frames averaged, and adjacent sections were separated by 126 μm. Images are obtainable using the Spectralis OCT system only when the quality score is >15; at lower scores the image acquisition process automatically stops and the image of the corresponding section is excluded. This study only included eyes with a quality score of >15 in all sections. The new software provided with the Spectralis OCT system automatically segmented the individual retinal layers, and the thicknesses of the mRNFL, GCIPL (calculated as the summation of the GCL and IPL thicknesses), and total retinal layer (TRL) were recorded for comparison with SS-OCT results. For SS-OCT, a 3D imaging data set was acquired for each subject with a raster scan protocol of 512 × 256 A-scans per data set. Each 3D scan covered an area of 7 mm × 7 mm centered on the fovea, and adjacent sections were separated by 27 μm. The segmentation algorithm of DRI SS-OCT provides the mRNFL, GCIPL, and TRL thicknesses.

The accuracy of the segmentation of each retinal layer (mRNFL, GCIPL, and TRL) and adequate centering on the fovea were reviewed independently by two masked observers (K.M.L. and E.J.L.). Only images that were considered adequate by both observers were included in the analysis.

**Fig 1:**
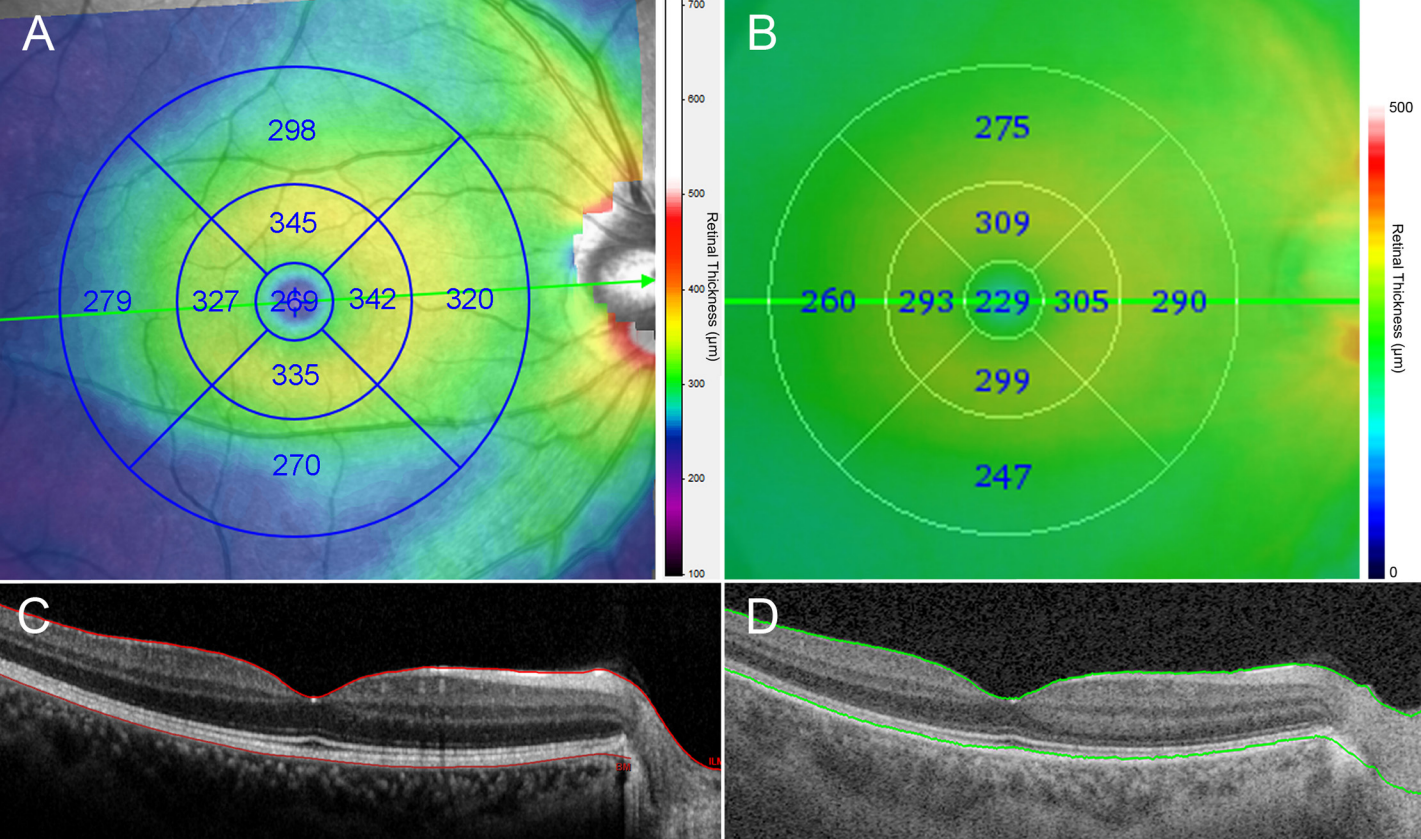
Retinal thickness maps showing mean total retinal layer thicknesses in each of the nine Early Treatment Diabetic Retinopathy Study (ETDRS) subfields as measured using spectral-domain optical coherence tomography (SD-OCT; A) and swept-source optical coherence tomography (SS-OCT; B). (C, D) B-scans obtained at the center of the macula (*green lines* in A and B, respectively). Note that the outer boundary is the retinal pigment epithelium in SD-OCT (*red line*, C), while it is the junction between the photoreceptor outer segment and retinal pigment epithelium in SS-OCT (*green line*, D).

In both types of OCT, we used retinal thickness map analysis to display numeric averages of the measurements for each of nine ETDRS subfields (Fig 1). The inner, intermediate, and outer rings with diameters of 1, 3, and 6 mm, respectively, were considered for the analysis. The average thicknesses of the following nine zones were used in the analysis: central fovea (CF), inner superior (IS), inner nasal (IN), inner inferior (II), inner temporal (IT), outer superior (OS), outer nasal (ON), outer inferior (OI), and outer temporal (OT) (Fig 1).

### Statistical analysis

**Fig 2:**
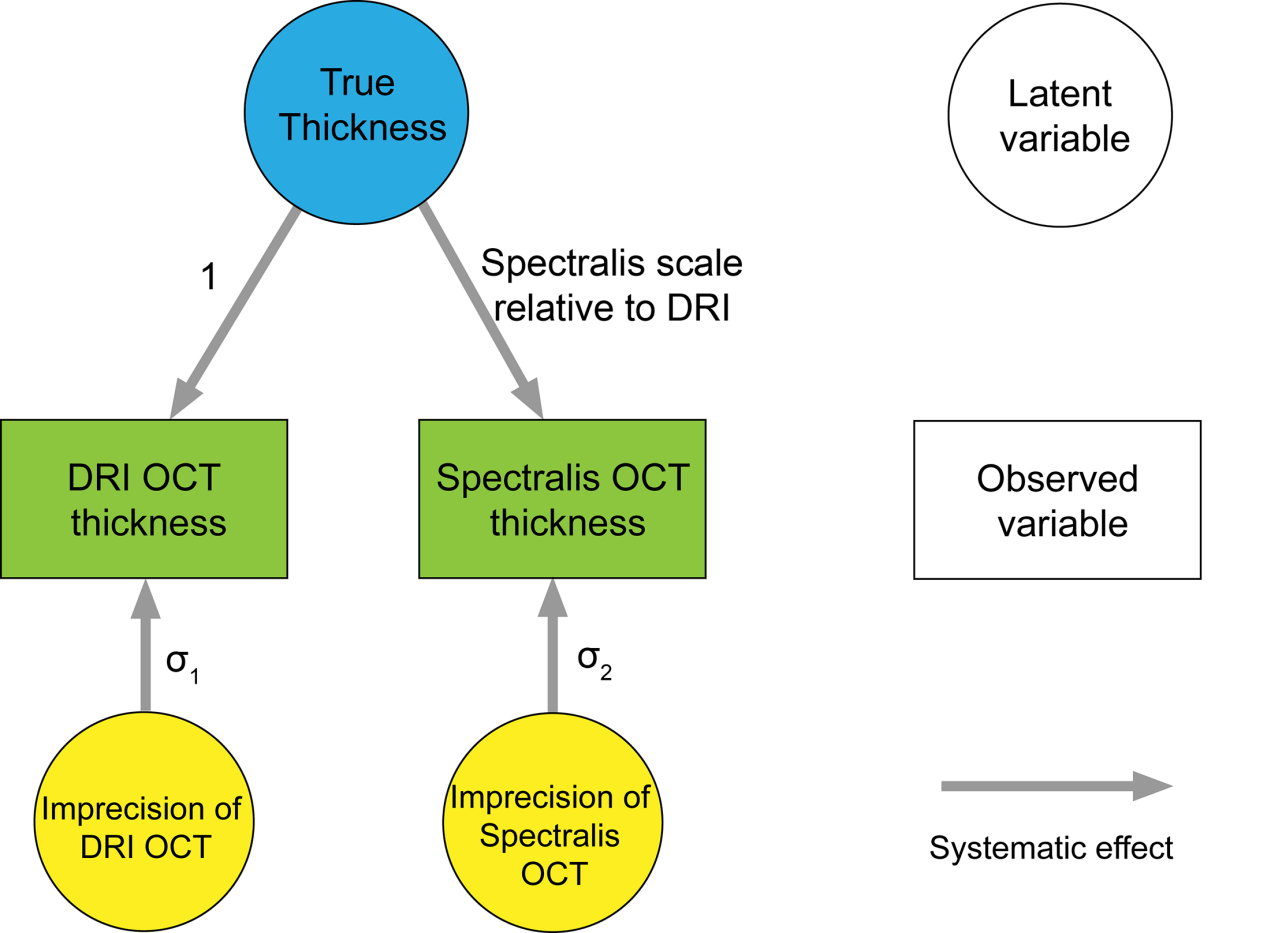
Path diagram of structural equation model (SEM) for comparison between the measurements from DRI SS-OCT and Spectralis SD-OCT. Scale represents relative bias.

Measurements recorded by a device reflect a combination of the true value (which is unknown) plus device-specific systematic error (relative bias) and a random error (imprecision). To account for the systematic and random errors present in each of the two devices, structural equation model (SEM) was used, and calibration equations, bias and imprecision of the measurements were calculated [28–30]. Fig 2 shows the path diagram illustrating the SEM that describes the relationship between the OCT measurements and the unknown true values. In the SEM, DRI SS-OCT measurement was chosen arbitrarily as the reference (path coefficient fixed to 1), and the method of full information maximum likelihood (FIML) was used to estimate the SEM parameters.

The measured mRNFL, GCIPL, and TRL thicknesses were compared between the two OCT systems using the paired *t*-test. The POAG and control groups were compared using the independent *t*-test. The threshold for statistical significance was set at *p*<0.05. Bonferroni correction was applied to raw data in the comparison of the subfield thicknesses and the areas under the receiver operating characteristics (ROC) curves (AUCs) on the basis of the number of comparisons within each analysis. The diagnostic ability of each measurement to differentiate between groups was determined by calculating the AUC. The ROC curve shows the trade-off between sensitivity and specificity. An AUC of 1.0 represents perfect discrimination, whereas an AUC of 0.5 represents chance discrimination. The following established five-category rating scale was used for interpreting AUC values: >0.90, excellent; 0.80–0.90, good; 0.70–0.80, fair; 0.60–0.70, poor; and 0.50–0.60, fail [31]. The statistical significance of the differences in the AUCs between the paired ROC curves of each system was calculated [32]. ROC regression analysis was performed using a probit model by maximum likelihood estimation to adjust the covariate effects of age, refractive error, gender, and glaucoma severity [33,34].

Statistical tests were performed with commercially available software (Stata version 13.0, StataCorp, College Station, TX, USA) and the OpenMx SEM package for the R language for statistical analysis to compute FIML estimates and likelihood-based confidence intervals. Except where indicated otherwise, the data are presented as mean±standard deviation values.

## Results

Sixty-five POAG eyes and 65 control eyes were initially enrolled. Of these, five eyes (three POAG eyes and two control eyes) were excluded due to poor image quality, and five eyes (two POAG eyes and three control eyes) were excluded during the age matching, leaving a final study population of 60 POAG eyes and 60 control eyes. The demographic and clinical characteristics of the POAG and control groups are summarized in Table 1.

**Table 1:** Demographic characteristics of primary open-angle glaucoma (POAG) and control groups.

	POAG (*n* = 60)	Control (*n* = 60)	*p*
Age (years)	59.9±11.5	60.4±13.5	0.833*
Gender (male:female)	30:30	23:37	0.198^†^
Refractive errors (diopters)	-0.88±2.55	-0.54±2.45	0.454^*^
IOP at examination (mmHg)	12.3±2.2	13.3±2.5	0.022*
Visual field mean deviation (dB)	-5.45±4.78	-0.35±1.40	<0.001*
SD-OCT global RNFL thickness (μm)	76.6±15.1	99.7±12.2	<0.001*
Central corneal thickness (μm)	549.1±31.8	555.8±28.2	0.230*
Axial length (mm)	23.9±1.4	23.6±1.5	0.252*

Data are mean±standard deviation values unless noted otherwise.

^*^ Independent *t*-test

^†^ χ^2^ test

Values those were significant are shown in bold.

IOP, intraocular pressure;

SD-OCT, spectral-domain optical coherence tomography

RNFL, retinal nerve fiber layer.

The TRL was thicker when measured using SD-OCT than using SS-OCT in all ETDRS subfields, with the thickness ratio between the SD-OCT and SS-OCT measurements being almost constant among all nine subfields (Table 2). This was because Spectralis SD-OCT set the outer boundary as the retinal pigment epithelium while DRI SS-OCT determined it as the junction between the photoreceptor outer segment and the retinal pigment epithelium (Fig 1).

**Table 2:** Total retinal layer (TRL) thicknesses in the 9 subfields between POAG and control groups.

TRL (μm)	POAG group				Control group				POAG vs Control (*p*^†^)	
	SD-OCT	SS-OCT	p^*^	SD/SS ratio	SD-OCT	SS-OCT	p^*^	SD/SS ratio	SD-OCT	SS-OCT
CF	261.65±18.28	220.73±17.81	**<0.001**	1.19±0.03	265.63±20.91	222.47±19.87	**<0.001**	1.20±0.03	0.269	0.616
IS	332.87±16.73	295.83±16.16	**<0.001**	1.13±0.02	336.08±15.05	298.17±15.16	**<0.001**	1.13±0.02	0.271	0.416
IN	337.10±16.07	297.80±15.62	**<0.001**	1.13±0.02	338.30±15.78	297.85±16.86	**<0.001**	1.14±0.03	0.681	0.987
II	324.05±19.33	289.77±17.96	**<0.001**	1.12±0.01	333.37±15.58	297.22±15.67	**<0.001**	1.12±0.02	**0.004**	0.017
IT	316.85±16.14	281.20±15.85	**<0.001**	1.13±0.02	325.08±14.44	289.03±14.85	**<0.001**	1.13±0.02	**0.004**	0.006
OS	285.27±19.38	260.85±19.01	**<0.001**	1.09±0.02	293.68±13.06	268.15±13.95	**<0.001**	1.10±0.02	0.006	0.018
ON	304.95±15.43	277.68±15.88	**<0.001**	1.10±0.02	311.38±16.11	281.82±15.80	**<0.001**	1.11±0.02	0.027	0.156
OI	262.72±13.48	241.30±15.31	**<0.001**	1.09±0.03	278.35±15.20	255.32±15.48	**<0.001**	1.09±0.02	**<0.001**	**<0.001**
OT	267.05±13.19	246.28±14.60	**<0.001**	1.09±0.03	276.78±11.76	255.87±12.97	**<0.001**	1.08±0.03	**<0.001**	**<0.001**

^*^ Comparisons were performed with the paired *t*-test.

^†^ Comparisons were performed with the independent *t*-test.

Values those were significant after Bonferroni correction (*p*≤0.0055; 0.05/9) are shown in bold.

SD-OCT, spectral-domain optical coherence tomography

SS-OCT, swept-source optical coherence tomography

CF, central fovea

IS, inner superior

IN, inner nasal

II, inner inferior

IT, inner temporal

OS, outer superior

ON, outer nasal

OI, outer inferior

OT, outer temporal.

The GCIPL was thicker in the inner zones and thinner in the CF and outer zones when measured using SD-OCT than when measured using SS-OCT (Table 3). The differences between the systems were significant in all of the subfields except for the IT zone in the POAG group (Table 3).

**Table 3:** Ganglion cell and inner plexus layer (GCIPL) thicknesses in the 9 subfields between POAG and control groups.

GCIPL (μm)	POAG group				Control group				POAG vs Control (*p*^†^)	
	SD-OCT	SS-OCT	p*	SD/SS ratio	SD-OCT	SS-OCT	p*	SD/SS ratio	SD-OCT	SS-OCT
CF	31.37±5.40	36.60±6.86	**<0.001**	0.87±0.12	33.50±6.50	38.37±6.79	**<0.001**	0.87±0.08	0.053	0.159
IS	83.50±11.98	80.93±11.73	**<0.001**	1.03±0.04	89.38±7.91	86.57±8.11	**<0.001**	1.03±0.03	**0.002**	**0.003**
IN	85.82±10.86	81.53±11.04	**<0.001**	1.05±0.04	88.70±9.11	83.58±9.30	**<0.001**	1.06±0.05	0.118	0.273
II	78.23±14.14	76.28±12.69	**<0.001**	1.02±0.05	89.12±7.78	86.18±8.33	**<0.001**	1.04±0.04	**<0.001**	**<0.001**
IT	73.52±12.35	72.38±10.68	0.075	1.01±0.07	85.45±8.35	81.60±7.54	**<0.001**	1.05±0.04	**<0.001**	**<0.001**
OS	56.22±9.07	60.17±9.75	**<0.001**	0.94±0.05	62.22±5.41	67.58±6.95	**<0.001**	0.92±0.04	**<0.001**	**<0.001**
ON	63.55±6.07	68.82±6.81	**<0.001**	0.92±0.04	67.78±6.94	73.80±8.52	**<0.001**	0.92±0.06	**0.001**	**0.001**
OI	48.47±5.33	53.73±6.76	**<0.001**	0.91±0.06	55.62±5.68	63.55±7.37	**<0.001**	0.88±0.06	**<0.001**	**<0.001**
OT	54.23±7.33	60.45±8.14	**<0.001**	0.90±0.06	65.82±6.44	70.23±6.74	**<0.001**	0.94±0.03	**<0.001**	**<0.001**

* Comparisons were performed with the paired *t*-test.

^†^ Comparisons were performed with the independent *t*-test.

Values those were significant after Bonferroni correction (*p*≤0.0055; 0.05/9) are shown in bold.

SD-OCT, spectral-domain optical coherence tomography

SS-OCT, swept-source optical coherence tomography

CF, central fovea

IS, inner superior

IN, inner nasal

II, inner inferior

IT, inner temporal

OS, outer superior

ON, outer nasal

OI, outer inferior

OT, outer temporal.

The mRNFL thickness differed significantly between SD-OCT and SS-OCT only in the central and ON zones in the POAG group, which were larger when measured with SD-OCT (Table 4). In the control group, the difference in the mRNFL thickness between the systems was significant in six subfields (CF, IT, and all four outer zones): except for the IT and OT zones, the SD-OCT measurements were larger than the SS-OCT measurements (Table 4).

**Table 4:** Macular RNFL (mRNFL) thicknesses in the 9 subfields between POAG and control groups.

mRNFL (μm)	POAG group				Control group				POAG vs Control (*p*^†^)	
	SD-OCT	SS-OCT	p*	SD/SS ratio	SD-OCT	SS-OCT	p*	SD/SS ratio	SD-OCT	SS-OCT
CF	10.90±2.30	3.42±2.30	**<0.001**	4.75±2.85	11.82±2.63	3.63±2.60	**<0.001**	4.79±3.09	0.045	0.630
IS	24.02±3.78	24.48±2.90	0.220	0.98±0.12	24.47±2.71	24.30±3.27	0.709	1.02±0.16	0.455	0.746
IN	21.77±2.45	21.88±2.08	0.679	1.00±0.10	21.48±1.93	21.52±2.98	0.937	1.02±0.19	0.483	0.436
II	24.08±3.90	24.08±4.10	0.999	1.03±0.29	26.45±3.15	25.08±3.48	0.016	1.08±0.21	**<0.001**	0.152
IT	19.02±1.53	18.25±3.76	0.119	1.12±0.44	18.95±1.41	20.45±2.54	**0.001**	0.94±0.16	0.805	**<0.001**
OS	33.97±9.05	34.23±6.88	0.566	0.98±0.11	39.10±5.58	37.32±4.25	**<0.001**	1.05±0.11	**<0.001**	**0.004**
ON	43.68±7.96	40.10±6.34	**<0.001**	1.09±0.08	48.22±7.43	41.40±5.68	**<0.001**	1.17±0.13	**0.002**	0.239
OI	28.55±7.69	28.27±8.57	0.666	1.11±0.53	40.42±7.44	37.73±5.26	**<0.001**	1.07±0.13	**<0.001**	**<0.001**
OT	19.05±1.76	18.37±4.76	0.295	1.13±0.41	20.45±1.87	23.02±3.27	**<0.001**	0.91±0.18	**<0.001**	**<0.001**

* Comparisons were performed with the paired *t*-test.

^†^ Comparisons were performed with the independent *t*-test.

Values those were significant after Bonferroni correction (*p*≤0.0055; 0.05/9) are shown in bold.

SD-OCT, spectral-domain optical coherence tomography

SS-OCT, swept-source optical coherence tomography

CF, central fovea

IS, inner superior

IN, inner nasal

II, inner inferior

IT, inner temporal

OS, outer superior

ON, outer nasal

OI, outer inferior

OT, outer temporal.

**Fig 3:**
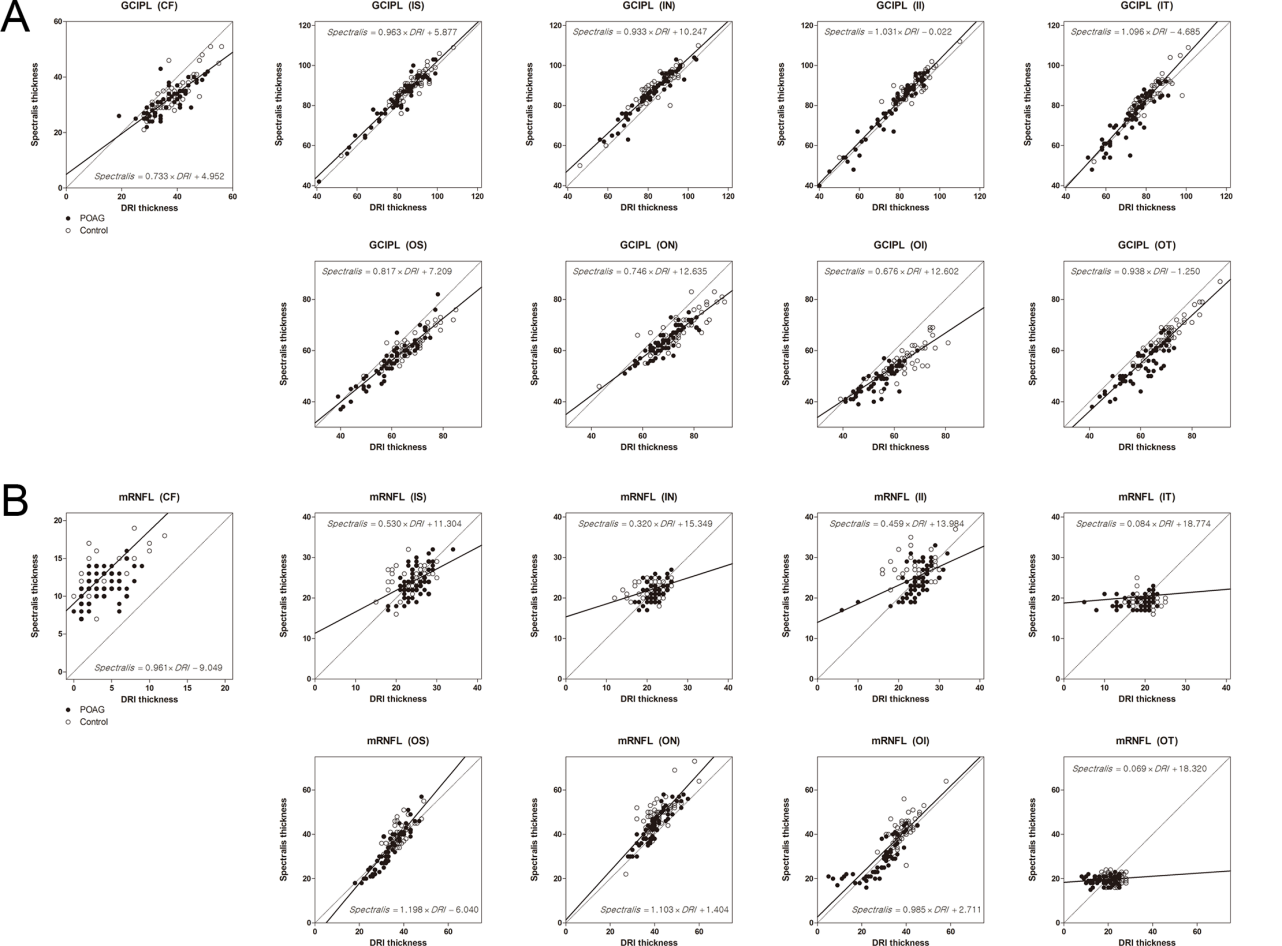
Calibration plots (*solid lines*) of the ganglion cell layer plus inner plexiform layer (GCIPL) (A) and macular retinal nerve fiber layer (mRNFL) (B) thicknesses (μm) for DRI SS-OCT and Spectralis SD-OCT. *Dotted lines* indicate the 45-degree reference lines of equality. POAG, primary open-angle glaucoma; CF, central fovea; IS, inner superior; IN, inner nasal; II, inner inferior; IT, inner temporal; OS, outer superior; ON, outer nasal; OI, outer inferior; OT, outer temporal.

Calibration equations between the two devices obtained based on the SEM parameter estimates and the calibration curves are shown in Fig 3. Using DRI SS-OCT as a reference for bias estimates, scale biases of Spectralis SD-OCT GCIPL thickness measurements were 0.733 (95% CI = 0.645–0.821) in CF, 0.963 (95% CI = 0.909–1.018) in IS, 0.933 (95% CI = 0.875–0.991) in IN, 1.031 (95% CI = 0.978–1.083) in II, 1.096 (95% CI = 1.020–1.172) in IT, 0.817 (95% CI = 0.762–0.871) in OS, 0.746 (95% CI = 0.675–0.818) in ON, 0.676 (95% CI = 0.612–0.741) in OI, and 0.938 (95% CI = 0.871–1.004) in OT (Fig 3A). Scale biases of Spectralis SD-OCT mRNFL thickness measurements were 0.961 (95% CI = –1.214–3.137) in CF, 0.530 (95% CI = 0.364–0.697) in IS, 0.320 (95% CI = 0.136–0.503) in IN, 0.459 (95% CI = 0.304–0.613) in II, 0.084 (95% CI = –13.299–13.466) in IT, 1.198 (95% CI = 1.088–1.309) in OS, 1.103 (95% CI = 0.967–1.238) in ON, 0.985 (95% CI = 0.881–1.088) in OI, and 0.069 (95% CI = –0.004–0.142) in OT (Fig 3B).

The glaucoma-diagnosis ability of each macular parameter was assessed and compared between the two OCT systems using AUCs (Table 5). For both SD-OCT and SS-OCT, good AUCs were found for the GCIPL thickness in the OI zone (AUC = 0.818 and 0.847, respectively) and OT zone (AUC = 0.894 and 0.825, respectively) and for the mRNFL thickness in the OI zone (AUC = 0.859 and 0.852, respectively). The AUC of Spectralis SD-OCT was largest for the GCIPL thickness in the OT zone [AUC = 0.894, 95% confidence interval (CI) = 0.839–0.950], which was significantly larger than the AUC of DRI SS-OCT in the corresponding subfield (*p* = 0.003). For DRI SS-OCT, the AUC was largest for the mRNFL thickness in the OI zone (AUC = 0.852, 95% CI = 0.783–0.921), but it was only comparable to the AUC of Spectralis SD-OCT in the corresponding sector (*p* = 0.770). The AUCs were comparable between the two systems in most of the subfields, but Spectralis SD-OCT performed better than DRI SS-OCT for the mRNFL thickness in the ON zone (*p* = 0.001) and for the GCIPL thickness in the OT zone (*p* = 0.003; Table 5).

**Table 5:** AUC, sensitivity and specificity of the macular inner retinal layer parameters obtained by the SD-OCT and SS-OCT.

	SD-OCT					SS-OCT					p*
	AUC (95% CI)	Cut off value (μm)	Sensitivity (%)	Specificity (%)	Correctly classified (%)	AUC (95% CI)	Cut off value (μm)	Sensitivity (%)	Specificity (%)	Correctly classified (%)	
GCIPL											
CF	0.586 (0.483–0.688)	29	88.3	33.3	60.8	0.564 (0.460–0.667)	31	91.7	21.7	56.7	0.446
IS	0.652 (0.553–0.752)	82	91.7	38.3	65.0	0.641 (0.542–0.741)	79	93.3	31.7	62.5	0.617
IN	0.570 (0.466–0.674)	79	95.0	25.0	60.0	0.552 (0.447–0.656)	74	93.3	25.0	59.2	0.389
II	0.735 (0.645–0.825)	80	96.7	45.0	70.8	0.740 (0.652–0.828)	79	86.7	51.7	69.2	0.810
IT	0.782 (0.700–0.864)	77	91.7	55.0	73.3	0.757 (0.671–0.844)	78	75.0	65.0	70.0	0.223
OS	0.719 (0.625–0.812)	57	85.0	68.3	68.3	0.721 (0.629–0.813)	63	78.3	63.3	70.8	0.896
ON	0.676 (0.580–0.772)	64	76.7	55.0	65.8	0.686 (0.591–0.781)	73	61.7	70.0	65.8	0.696
OI	0.818 (0.744–0.892)	53	76.7	71.7	74.2	0.847 (0.777–0.916)	60	76.7	80.0	78.3	0.208
OT	**0.894 (0.839–0.950)**	63	75.0	90.0	82.5	**0.825 (0.752–0.897)**	67	76.7	73.3	75.0	**0.003**
mRNFL											
CF	0.582 (0.480–0.684)	15	20.0	96.7	58.3	0.519 (0.415–0.622)	2	78.3	30.0	54.2	0.159
IS	0.552 (0.446–0.658)	23	80.0	40.0	60.0	0.516 (0.412–0.620)	25	51.7	58.3	55.0	0.510
IN	0.471 (0.367–0.575)	20	81.7	23.3	52.5	0.509 (0.405–0.613)	23	40.0	66.7	53.3	0.528
II	0.676 (0.578–0.775)	23	96.7	40.0	68.3	0.584 (0.481–0.686)	25	63.3	53.3	58.3	0.094
IT	0.486 (0.383–0.589)	18	91.7	20.0	55.8	0.675 (0.580–0.770)	21	53.3	73.3	63.3	0.008
OS	0.673 (0.574–0.772)	34	91.7	50.0	70.8	0.637 (0.535–0.740)	34	85.0	48.3	66.7	0.162
ON	**0.659 (0.560–0.759)**	41	91.7	38.3	65.0	**0.555 (0.451–0.659)**	36	93.3	23.3	58.3	**0.001**
OI	0.859 (0.792–0.925)	36	81.7	81.7	81.7	0.852 (0.783–0.921)	35	81.7	78.3	80.0	0.770
OT	0.713 (0.622–0.804)	20	71.7	63.3	67.5	0.784 (0.705–0.864)	22	71.7	71.7	71.7	0.246

* Comparison between AUCs. Values with significant difference after Bonferroni correction (*p*≤0.0055; 0.05/9) are shown in bold.

AUC, area under the receiver operating curve

SD-OCT, spectral-domain optical coherence tomography

SS-OCT, swept-source optical coherence tomography

CI, confidence interval

GCIPL, ganglion cell inner plexus layer

mRNFL, macular retinal nerve fiber layer

CF, central fovea

IS, inner superior

IN, inner nasal

II, inner inferior

IT, inner temporal

OS, outer superior

ON, outer nasal

OI, outer inferior

OT, outer temporal.

**Fig 4:**
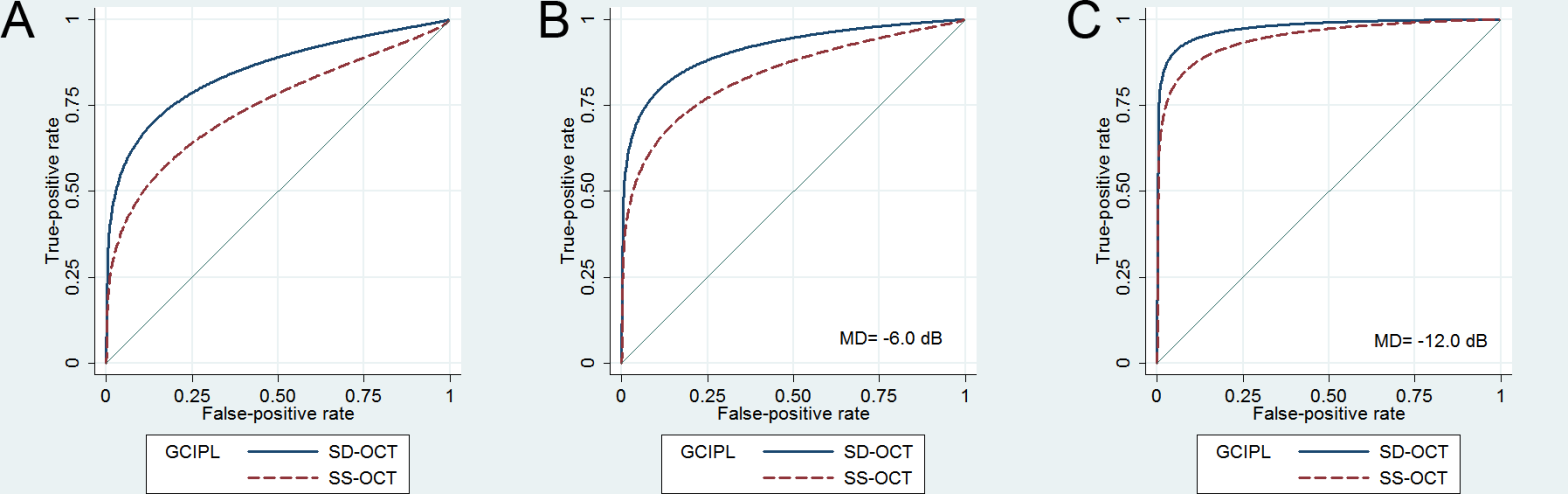
Receiver operating characteristic (ROC) curves of the GCIPL thickness in the outer temporal zone obtained using the ROC regression model by a generalized linear regression model. ROC curve obtained using the ROC regression model (**A**), and that obtained at fixed visual field mean deviations (MDs) of –6.0 dB (**B**), and –12.0 dB (**C**). Although the difference in the area under the ROC curve (AUC) between the two systems decreases as MD decreases, the AUCs of SD-OCT remain larger than those of SS-OCT.

An ROC regression model was applied to determine the factors affecting the glaucoma-diagnosis ability for the GCIPL thickness in the OT zone; the factors analyzed were age, gender, spherical error, visual field mean deviation, and the type of OCT. The regression model revealed that a larger AUC was associated with a smaller visual field mean deviation and the use of Spectralis SD-OCT (Table 6). Spectralis SD-OCT was better than DRI SS-OCT for measuring the GCIPL thickness in the OT zone irrespective of the degree of visual field damage (Fig 4).

**Table 6:** Results of the ROC regression model incorporating age, refractive error, disease severity, and type of OCT as covariates in the outer temporal zone.

Covariates	GCIPL			POAG	Control
	β	95% CI	*p*	p*	*p*^†^
Age	0.012	(-0.022, 0.046)	0.490	0.489	0.420
Female	0.078	(-0.568, 0.724)	0.813	0.813	0.410
Sph	-0.082	(-0.223, 0.060)	0.258	0.258	0.749
VF MD	-0.124	(-0.192, -0.057)	**<0.001**	**<0.001**	
SD-OCT^‡^	0.435	(0.263, 0.607)	**<0.001**	**<0.001**	**<0.001**

* Effects of the covariates on the GCIPL thickness in the case and control population.

^†^ Coefficient of the SD-OCT over the SS-OCT.

Significant values (*p*<0.05) using the ROC regression analysis by a generalized linear regression model are shown in bold.

ROC, receiver operating characteristics

GCIPL, ganglion cell inner plexus layer

CI, confidence interval

Sph, spherical equivalent (diopters)

VF, visual field

MD, mean deviation (dB).

## Discussion

The present study compared the diagnostic abilities of Spectralis SD-OCT- and DRI SS-OCT-based macular inner layer analysis to differentiate glaucomatous eyes from control eyes. The results showed that although the diagnostic abilities of the two OCT systems were comparable in most of the macular subfields, Spectralis SD-OCT was better in some areas. To our knowledge, this is the first study to compare the ability of new segmentation software for Spectralis SD-OCT in the macular area with that of DRI SS-OCT.

While the agreement between the two systems was generally good (Fig 3), there was an inherent difference in the measurement of the macular retinal layer thickness between the two OCT systems. Firstly, TRL was universally thicker when measured using SD-OCT than SS-OCT. Secondly, GCIPL was thicker in the inner zones while the other zones were thinner for SD-OCT compared to SS-OCT. Thirdly, mRNFL thickness differences between the systems varied among sectors, but SD-OCT generally produced thicker measurements. The intersystem differences in the TRL thicknesses were due to the difference in the inherent segmentation algorithm between SD-OCT and SS-OCT. However, we currently do not have clear answers for the source of discrepancy in the GCIPL and mRNFL thicknesses between the systems, and which system would provide more accurate measurements. Several postulations could be made based on the findings of this study, as described below.

In some GCIPL (IT; Table 3) and mRNFL (IT, OS, OI and OT; Table 4) subfields, the significant differences between the two systems that were evident in the control group were not present in the POAG group. The pattern was also discernible in the calibration curves (see Fig 3), where the thickness differences between the two systems were generally larger in the control group than in the POAG group. This finding may indicate that the DRI SS-OCT underestimated the loss of RNFL or GCL, thereby producing measurements in which the RNFL or GCL in the damaged area was thicker than for the Spectralis SD-OCT, which may have reduced the inherent discrepancy between the OCT systems. The low detectability of the damaged inner retinal layers using DRI SS-OCT may be attributable to several factors. Firstly, DRI SS-OCT uses longer wavelength, which penetrates deeper into the tissue [35–38]. Although this deeper tissue penetration of SS-OCT may facilitate the examination of deep tissues [35–38], the longer wavelength also means that the resolution is not necessarily sufficiently higher in the inner retinal tissues. We speculate that the lower resolution of the inner retina in DRI SS-OCT images may have resulted in a lower accuracy in the inner retinal layer segmentation, and so reduced the possibility of detection of the thinned area. Secondly, Spectralis SD-OCT involves averaging nine OCT frames for a single B-scan image, while DRI SS-OCT does not involve averaging multiple images but uses denser sampling than that in Spectralis SD-OCT [39]. Although this denser sampling potentially yields images of higher quality, the averaging of multiple images in the Spectralis system may enhance the image contrast and thereby facilitate segmentation of the inner retinal layer. In terms of indicating the presence of glaucoma, less-dense sampling with a higher signal-to-noise ratio (in the Spectralis system) may provide better stability of the segmentation compared to denser sampling with the associated likelihood of a higher variability of the measurements (in the DRI-OCT1 Atlantis system). In addition, the eye-tracking functionality of the Spectralis system may reduce motion artifacts in the course of acquiring multiple images [40]. Thirdly, it is also possible that the less-precise segmentation of SS-OCT was attributable to the intrinsic segmentation software of the DRI system.

Despite the potential superiority of Spectralis SD-OCT in segmentation of the inner retinal layer, Spectralis SD-OCT and DRI SS-OCT exhibited very similar glaucoma-diagnosis abilities. However, it is noteworthy that the AUC was largest for the GCIPL thickness in the OT zone as measured by SD-OCT, and significantly larger than the AUC of SS-OCT in the corresponding zone. ROC regression analysis also showed that measurements of the GCIPL thickness in the OT zone were better for SD-OCT than for SS-OCT, irrespective of the degree of visual field damage. This is suggestive of the superiority of Spectralis SD-OCT over DRI SS-OCT in the evaluation of the GCIPL, because the OT zone of the macula is one with the earliest sites of GCIPL damage in glaucoma [41].

In the present study, the AUCs were largest in the OT and OI zones for both SD-OCT and SS-OCT. The better diagnosis ability when using the outer zones than the inner zones is consistent with the results of previous studies [13,42]. The inferior arcuate fibers followed by the superior arcuate fibers are known to be the most susceptible to glaucomatous damage, while papillomacular fibers are generally the last to be involved in glaucoma [43]. Therefore, it is relevant that glaucomatous damage is more likely to be detected in the outer macular zones. On the other hand, the better diagnosis ability when using the inferior and temporal zones than the superior or nasal zones may be explained by the different distribution of the superior and inferior arcuate fibers. Most RGCs in the inferior macular region project their axons to the inferior optic nerve head (ONH) quadrant [44]. Therefore, glaucomatous damage at the inferior pole of the ONH may result in damage to the inferior and inferotemporal macular zones [41]. However, RGCs in the superior macular region project axons to the temporal ONH quadrant [44], which is generally not involved until end stage of disease [43]. Thus, significant thinning would not be detected in the superior macular zone. Meanwhile, axons entering the superior ONH quadrant are originated from the area which is not included in the mGCL analysis. Thus, axonal damage occurring at the superior pole of the ONH may not be detected in the macular analysis [41].

On the other hand, the location with the best AUC differed between the mRNFL and GCIPL parameters: only the OI zone had a good AUC for the mRNFL thickness parameters, while both the OI and OT zones showed good AUCs for GCIPL thicknesses; this may be attributable to the spatial discrepancy between the axons and their original RGCs [41,45]: Cell bodies located at the temporal retina project their axons mainly through the inferior retina, and so mRNFL damage in the OI zone may be associated with damage to the GCL in the OT zone. Meanwhile, it is also possible that the different topographies of the RNFL and GCL in the macular area influence the ability to detect thinning in each macular zone [46]: the mRNFL is originally thinner in the OT zone than in other macular zones, and thus thinning of the mRNFL in this area might not have been detected by the segmentation algorithm.

The present study was limited by several factors. Firstly, there were inherent differences in the segmentation algorithm between Spectralis SD-OCT and DRI SS-OCT. Other than the difference in the definition of the TRL, the method to obtain the GCIPL differed between systems: Spectralis SD-OCT measured the GCL and IPL separately, while in DRI SS-OCT the GCIPL measured GCL and IPL together as a single parameter. Secondly, neither of the two OCT devices provides a normative database of macular inner layer thicknesses, and so the diagnostic performance was evaluated based on the AUC. Thirdly, the AUC values presented in this study indicate that OCT macular inner retinal layer analysis alone might not be an adequate technique for glaucoma diagnosis; that is, while it could be a useful adjunct for evaluating the glaucomatous damage of the macula, it might not replace conventional methods in the diagnosis of glaucoma. Forthly, the thickness measurement can be influenced by various factors, such as the direction of the light source or the head posture of the patient. An oblique light source could result in overestimation [47], and cyclotorsion of the fundus may result in a different allocation of the mean thickness in each ETDRS subfield. Lastly, the anatomical orientation specific to each eye, such as the fovea–Bruch’s membrane opening plane [48,49] or the direction of the temporal raphe [50], were not considered in the ETDRS subfield analysis. Although the Spectralis OCT system evaluates the macular thickness based on the fovea–Bruch’s membrane opening plane, this concept is not incorporated in the DRI-OCT1 Atlantis system, and so ETDRS subfield based analysis, which does not consider the fovea–Bruch’s membrane opening plane was chosen in both systems for the comparison.

In conclusion, the ability to diagnose glaucoma based on analyzing the macular inner layer thickness was largely comparable between Spectralis SD-OCT and DRI SS-OCT. The AUC for diagnosing glaucoma was largest for the GCIPL thickness in the OT zone as measured by Spectralis SD-OCT. This is the zone where glaucomatous RGC damage occurs most frequently, and hence the present results suggest the potential superiority of Spectralis SD-OCT over DRI SS-OCT in diagnosing glaucoma in the macular region.

## References

[1] Weinreb RN, Aung T, Medeiros FA. The pathophysiology and treatment of glaucoma: a review. JAMA. 2014;311(18):1901–1911. Epub 2014/05/16. 10.1001/jama.2014.3192 . 24825645 PMC4523637

[2] Danias J, Shen F, Kavalarakis M, Chen B, Goldblum D, Lee K, et al Characterization of retinal damage in the episcleral vein cauterization rat glaucoma model. Exp Eye Res. 2006;82(2):219–228. 10.1016/j.exer.2005.06.013 16109406 PMC1401487

[3] Huang D, Swanson EA, Lin CP, Schuman JS, Stinson WG, Chang W, et al Optical coherence tomography. Science. 1991;254(5035):1178–1181. . 1957169 10.1126/science.1957169 PMC4638169

[4] Schuman JS, Pedut-Kloizman T, Hertzmark E, Hee MR, Wilkins JR, Coker JG, et al Reproducibility of nerve fiber layer thickness measurements using optical coherence tomography. Ophthalmology. 1996;103(11):1889–1898. 8942887 10.1016/s0161-6420(96)30410-7 PMC1939724

[5] Medeiros FA, Zangwill LM, Bowd C, Vessani RM, Susanna R, Jr., Weinreb RN. Evaluation of retinal nerve fiber layer, optic nerve head, and macular thickness measurements for glaucoma detection using optical coherence tomography. Am J Ophthalmol. 2005;139(1):44–55. 10.1016/j.ajo.2004.08.069 . 15652827

[6] Wollstein G, Schuman JS, Price LL, Aydin A, Beaton SA, Stark PC, et al Optical coherence tomography (OCT) macular and peripapillary retinal nerve fiber layer measurements and automated visual fields. Am J Ophthalmol. 2004;138(2):218–225. 10.1016/j.ajo.2004.03.019 . 15289130

[7] Lederer DE, Schuman JS, Hertzmark E, Heltzer J, Velazques LJ, Fujimoto JG, et al Analysis of macular volume in normal and glaucomatous eyes using optical coherence tomography. Am J Ophthalmol. 2003;135(6):838–843. . 12788124 10.1016/s0002-9394(02)02277-8

[8] Kim NR, Lee ES, Seong GJ, Kim JH, An HG, Kim CY. Structure-function relationship and diagnostic value of macular ganglion cell complex measurement using Fourier-domain OCT in glaucoma. Invest Ophthalmol Vis Sci. 2010;51(9):4646–4651. Epub 2010/05/04. 10.1167/iovs.09-5053 . 20435603

[9] Huang JY, Pekmezci M, Mesiwala N, Kao A, Lin S. Diagnostic power of optic disc morphology, peripapillary retinal nerve fiber layer thickness, and macular inner retinal layer thickness in glaucoma diagnosis with fourier-domain optical coherence tomography. J Glaucoma. 2011;20(2):87–94. Epub 2010/06/26. 10.1097/IJG.0b013e3181d787b6 . 20577117

[10] Tan O, Chopra V, Lu AT, Schuman JS, Ishikawa H, Wollstein G, et al Detection of macular ganglion cell loss in glaucoma by Fourier-domain optical coherence tomography. Ophthalmology. 2009;116(12):2305–2314 e2301-2302. Epub 2009/09/12. 10.1016/j.ophtha.2009.05.025 19744726 PMC2787911

[11] Hwang YH, Jeong YC, Kim HK, Sohn YH. Macular ganglion cell analysis for early detection of glaucoma. Ophthalmology. 2014;121(8):1508–1515. Epub 2014/04/08. 10.1016/j.ophtha.2014.02.019 . 24702756

[12] Tan O, Li G, Lu AT, Varma R, Huang D, Advanced Imaging for Glaucoma Study G. Mapping of macular substructures with optical coherence tomography for glaucoma diagnosis. Ophthalmology. 2008;115(6):949–956. 10.1016/j.ophtha.2007.08.011 17981334 PMC2692598

[13] Nakano N, Hangai M, Nakanishi H, Mori S, Nukada M, Kotera Y, et al Macular ganglion cell layer imaging in preperimetric glaucoma with speckle noise-reduced spectral domain optical coherence tomography. Ophthalmology. 2011;118(12):2414–2426. Epub 2011/09/20. 10.1016/j.ophtha.2011.06.015 . 21924499

[14] Nouri-Mahdavi K, Nowroozizadeh S, Nassiri N, Cirineo N, Knipping S, Giaconi J, et al Macular ganglion cell/inner plexiform layer measurements by spectral domain optical coherence tomography for detection of early glaucoma and comparison to retinal nerve fiber layer measurements. Am J Ophthalmol. 2013;156(6):1297–1307 e1292. 10.1016/j.ajo.2013.08.001 24075422 PMC3834195

[15] Mwanza JC, Oakley JD, Budenz DL, Chang RT, Knight OJ, Feuer WJ. Macular ganglion cell-inner plexiform layer: automated detection and thickness reproducibility with spectral domain-optical coherence tomography in glaucoma. Invest Ophthalmol Vis Sci. 2011;52(11):8323–8329. 10.1167/iovs.11-7962 21917932 PMC3208140

[16] Sung KR, Sun JH, Na JH, Lee JY, Lee Y. Progression detection capability of macular thickness in advanced glaucomatous eyes. Ophthalmology. 2012;119(2):308–313. 10.1016/j.ophtha.2011.08.022 . 22182800

[17] Na JH, Sung KR, Baek S, Kim YJ, Durbin MK, Lee HJ, et al Detection of glaucoma progression by assessment of segmented macular thickness data obtained using spectral domain optical coherence tomography. Invest Ophthalmol Vis Sci. 2012;53(7):3817–3826. 10.1167/iovs.11-9369 . 22562510

[18] Kim NR, Lee ES, Seong GJ, Kang SY, Kim JH, Hong S, et al Comparing the ganglion cell complex and retinal nerve fibre layer measurements by Fourier domain OCT to detect glaucoma in high myopia. Br J Ophthalmol. 2011;95(8):1115–1121. Epub 2010/09/02. 10.1136/bjo.2010.182493 . 20805125

[19] Choi YJ, Jeoung JW, Park KH, Kim DM. Glaucoma detection ability of ganglion cell-inner plexiform layer thickness by spectral-domain optical coherence tomography in high myopia. Invest Ophthalmol Vis Sci. 2013;54(3):2296–2304. 10.1167/iovs.12-10530 . 23462754

[20] Kimura Y, Hangai M, Matsumoto A, Akagi T, Ikeda HO, Ohkubo S, et al Macular structure parameters as an automated indicator of paracentral scotoma in early glaucoma. Am J Ophthalmol. 2013;156(5):907–917 e901. 10.1016/j.ajo.2013.06.029 . 23972895

[21] Shin HY, Park HY, Jung KI, Choi JA, Park CK. Glaucoma diagnostic ability of ganglion cell-inner plexiform layer thickness differs according to the location of visual field loss. Ophthalmology. 2014;121(1):93–99. 10.1016/j.ophtha.2013.06.041 . 23962652

[22] Yamada H, Hangai M, Nakano N, Takayama K, Kimura Y, Miyake M, et al Asymmetry analysis of macular inner retinal layers for glaucoma diagnosis. Am J Ophthalmol. 2014;158(6):1318–1329 e1313. 10.1016/j.ajo.2014.08.040 . 25194230

[23] Martinez-de-la-Casa JM, Cifuentes-Canorea P, Berrozpe C, Sastre M, Polo V, Moreno-Montanes J, et al Diagnostic ability of macular nerve fiber layer thickness using new segmentation software in glaucoma suspects. Invest Ophthalmol Vis Sci. 2014;55(12):8343–8348. Epub 2014/11/27. 10.1167/iovs.14-15501 . 25425301

[24] Miki A, Ikuno Y, Jo Y, Nishida K. Comparison of enhanced depth imaging and high-penetration optical coherence tomography for imaging deep optic nerve head and parapapillary structures. Clin Ophthalmol. 2013;7:1995–2001. 10.2147/OPTH.S50120 24133368 PMC3797242

[25] Park HY, Shin HY, Park CK. Imaging the posterior segment of the eye using swept-source optical coherence tomography in myopic glaucoma eyes: comparison with enhanced-depth imaging. Am J Ophthalmol. 2014;157(3):550–557. 10.1016/j.ajo.2013.11.008 . 24239773

[26] Yang Z, Tatham AJ, Zangwill LM, Weinreb RN, Zhang C, Medeiros FA. Diagnostic ability of retinal nerve fiber layer imaging by swept-source optical coherence tomography in glaucoma. Am J Ophthalmol. 2015;159(1):193–201. Epub 2014/12/03. 10.1016/j.ajo.2014.10.019 25448991 PMC4293127

[27] Yang Z, Tatham AJ, Weinreb RN, Medeiros FA, Liu T, Zangwill LM. Diagnostic ability of macular ganglion cell inner plexiform layer measurements in glaucoma using swept source and spectral domain optical coherence tomography. PLoS One. 2015;10(5):e0125957 Epub 2015/05/16. 10.1371/journal.pone.0125957 ; PubMed Central PMCID: PMCPmc4433247. 25978420 PMC4433247

[28] Buchser NM, Wollstein G, Ishikawa H, Bilonick RA, Ling Y, Folio LS, et al Comparison of retinal nerve fiber layer thickness measurement bias and imprecision across three spectral-domain optical coherence tomography devices. Invest Ophthalmol Vis Sci. 2012;53(7):3742–3747. Epub 2012/04/28. 10.1167/iovs.11-8432 ; PubMed Central PMCID: PMCPmc3390182. 22538423 PMC3390182

[29] Pierro L, Gagliardi M, Iuliano L, Ambrosi A, Bandello F. Retinal nerve fiber layer thickness reproducibility using seven different OCT instruments. Invest Ophthalmol Vis Sci. 2012;53(9):5912–5920. Epub 2012/08/09. 10.1167/iovs.11-8644 . 22871835

[30] Ferguson AA, Bilonick RA, Buchanich JM, Marsh GM, Fisher AL. How Well Do Raters Agree on the Development Stage of Caenorhabditis elegans? PLoS One. 2015;10(7):e0132365 Epub 2015/07/15. 10.1371/journal.pone.0132365 ; PubMed Central PMCID: PMCPmc4501796. 26172989 PMC4501796

[31] Swets JA. Measuring the accuracy of diagnostic systems. Science. 1988;240(4857):1285–1293. Epub 1988/06/03. . 3287615 10.1126/science.3287615

[32] Hanley JA, McNeil BJ. A method of comparing the areas under receiver operating characteristic curves derived from the same cases. Radiology. 1983;148(3):839–843. Epub 1983/09/01. 10.1148/radiology.148.3.6878708 . 6878708

[33] Dodd LE, Pepe MS. Partial AUC estimation and regression. Biometrics. 2003;59(3):614–623. . 14601762 10.1111/1541-0420.00071

[34] Medeiros FA, Sample PA, Zangwill LM, Liebmann JM, Girkin CA, Weinreb RN. A statistical approach to the evaluation of covariate effects on the receiver operating characteristic curves of diagnostic tests in glaucoma. Invest Ophthalmol Vis Sci. 2006;47(6):2520–2527. 10.1167/iovs.05-1441 . 16723465

[35] Matsuo Y, Sakamoto T, Yamashita T, Tomita M, Shirasawa M, Terasaki H. Comparisons of choroidal thickness of normal eyes obtained by two different spectral-domain OCT instruments and one swept-source OCT instrument. Invest Ophthalmol Vis Sci. 2013;54(12):7630–7636. Epub 2013/10/31. 10.1167/iovs.13-13135 . 24168999

[36] Adhi M, Liu JJ, Qavi AH, Grulkowski I, Lu CD, Mohler KJ, et al Choroidal analysis in healthy eyes using swept-source optical coherence tomography compared to spectral domain optical coherence tomography. Am J Ophthalmol. 2014;157(6):1272–1281.e1271. Epub 2014/02/25. 10.1016/j.ajo.2014.02.034 . 24561169

[37] Copete S, Flores-Moreno I, Montero JA, Duker JS, Ruiz-Moreno JM. Direct comparison of spectral-domain and swept-source OCT in the measurement of choroidal thickness in normal eyes. Br J Ophthalmol. 2014;98(3):334–338. Epub 2013/11/30. 10.1136/bjophthalmol-2013-303904 . 24288394

[38] Adhi M, Liu JJ, Qavi AH, Grulkowski I, Fujimoto JG, Duker JS. Enhanced visualization of the choroido-scleral interface using swept-source OCT. Ophthalmic Surg Lasers Imaging Retina. 2013;44(6 Suppl):S40–42. Epub 2013/11/28. 10.3928/23258160-20131101-08 . 24220884

[39] Potsaid B, Baumann B, Huang D, Barry S, Cable AE, Schuman JS, et al Ultrahigh speed 1050nm swept source/Fourier domain OCT retinal and anterior segment imaging at 100,000 to 400,000 axial scans per second. Opt Express. 2010;18(19):20029–20048. Epub 2010/10/14. 10.1364/oe.18.020029 ; PubMed Central PMCID: PMCPmc3136869. 20940894 PMC3136869

[40] Langenegger SJ, Funk J, Toteberg-Harms M. Reproducibility of retinal nerve fiber layer thickness measurements using the eye tracker and the retest function of Spectralis SD-OCT in glaucomatous and healthy control eyes. Invest Ophthalmol Vis Sci. 2011;52(6):3338–3344. Epub 2011/02/19. 10.1167/iovs.10-6611 . 21330656

[41] Hood DC, Raza AS, de Moraes CG, Liebmann JM, Ritch R. Glaucomatous damage of the macula. Prog Retin Eye Res. 2013;32:1–21. 10.1016/j.preteyeres.2012.08.003 22995953 PMC3529818

[42] Nakatani Y, Higashide T, Ohkubo S, Takeda H, Sugiyama K. Evaluation of macular thickness and peripapillary retinal nerve fiber layer thickness for detection of early glaucoma using spectral domain optical coherence tomography. J Glaucoma. 2011;20(4):252–259. Epub 2010/06/04. 10.1097/IJG.0b013e3181e079ed . 20520570

[43] Quigley HA, Addicks EM. Regional differences in the structure of the lamina cribrosa and their relation to glaucomatous optic nerve damage. Arch Ophthalmol. 1981;99(1):137–143. . 7458737 10.1001/archopht.1981.03930010139020

[44] Bagga H, Greenfield DS, Knighton RW. Macular symmetry testing for glaucoma detection. J Glaucoma. 2005;14(5):358–363. . 16148583 10.1097/01.ijg.0000176930.21853.04

[45] Lee K, Kwon YH, Garvin MK, Niemeijer M, Sonka M, Abramoff MD. Distribution of damage to the entire retinal ganglion cell pathway: quantified using spectral-domain optical coherence tomography analysis in patients with glaucoma. Arch Ophthalmol. 2012;130(9):1118–1126. 10.1001/archophthalmol.2012.669 22965586 PMC3691810

[46] Curcio CA, Messinger JD, Sloan KR, Mitra A, McGwin G, Spaide RF. Human chorioretinal layer thicknesses measured in macula-wide, high-resolution histologic sections. Invest Ophthalmol Vis Sci. 2011;52(7):3943–3954. Epub 2011/03/23. 10.1167/iovs.10-6377 ; PubMed Central PMCID: PMCPmc3175964. 21421869 PMC3175964

[47] Knighton RW, Qian C. An optical model of the human retinal nerve fiber layer: implications of directional reflectance for variability of clinical measurements. J Glaucoma. 2000;9(1):56–62. Epub 2000/03/09. . 10708233 10.1097/00061198-200002000-00011

[48] Chauhan BC, Burgoyne CF. From clinical examination of the optic disc to clinical assessment of the optic nerve head: a paradigm change. Am J Ophthalmol. 2013;156(2):218–227.e212. Epub 2013/06/19. 10.1016/j.ajo.2013.04.016 ; PubMed Central PMCID: PMCPmc3720683. 23768651 PMC3720683

[49] Amini N, Nowroozizadeh S, Cirineo N, Henry S, Chang T, Chou T, et al Influence of the disc-fovea angle on limits of RNFL variability and glaucoma discrimination. Invest Ophthalmol Vis Sci. 2014;55(11):7332–7342. Epub 2014/10/11. 10.1167/iovs.14-14962 ; PubMed Central PMCID: PMCPmc4235330. 25301880 PMC4235330

[50] Chauhan BC, Sharpe GP, Hutchison DM. Imaging of the temporal raphe with optical coherence tomography. Ophthalmology. 2014;121(11):2287–2288. 10.1016/j.ophtha.2014.06.023 . 25156139

